# Development and application of recombinase polymerase amplification assay for rapid detection of *Blastocystis* sp.

**DOI:** 10.1017/S0031182023000975

**Published:** 2023-11

**Authors:** Xuefang Mei, Changwei Su, Shanrui Zhang, Luwei Jia, Zhenke Yang, Xiaowei Tian, Zhenchao Zhang, Shuai Wang

**Affiliations:** Department of Pathogenic Biology, Xinxiang Key Laboratory of Pathogenic Biology, School of Basic Medical Sciences, Xinxiang Medical University, Xinxiang, Henan 453003, PR China

**Keywords:** application, *Blastocystis* sp., development, rapid detection, recombinase polymerase amplification

## Abstract

*Blastocystis* sp. is a common parasite in the intestinal tract of humans and animals. The clinical diagnosis of *Blastocystis* sp. mainly depends on the microscopic observation of parasite, which can lead to false-negative results. An accurate and convenient diagnostic approach for *Blastocystis* sp. infection is crucial for effectively preventing and controlling blastocystosis. Herein, we developed a recombinase polymerase amplification (RPA) method for detecting *Blastocystis* sp. The results showed that the DNA amplification by RPA established in this study could be performed within 5 min at 37°C, with maximum band intensity observed at 30 min. The minimum detection limit of RPA was 100 fg *μ*L^−1^, consistent with conventional polymerase chain reaction (cPCR). Furthermore, the RPA method exhibited no cross-reactivity with 7 other non-target pathogens in the intestinal tract. Next, the newly established RPA method was used to analyse 40 fecal samples collected clinically, and the detection results were consistent with cPCR. These results corroborate that the newly developed RPA method has good sensitivity and specificity and offers the advantage of short detection times, which can be harnessed for differential diagnosis and rapid detection of *Blastocystis* sp.

## Graphical abstract

**Figure:**
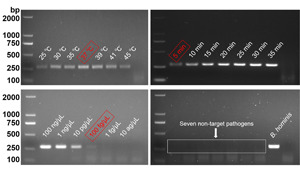

## Introduction

Diarrhoea caused by parasitic protozoa is the second leading cause of death among children worldwide (Kotloff *et al*., 2013). In most cases, the specific cause of diarrhoea remains unidentified, which can be caused by various intestinal pathogens, including viruses, bacteria and protozoan parasites (Caner *et al*., 2020; David *et al*., 2022; Kotloff, 2022). Acute diarrhoea is mainly caused by viral infections, while persistent chronic diarrhoea is more commonly caused by parasitic infections, which are increasingly recognized as the main cause of diarrhoeal diseases (Lima and Guerrant, 1992). It has been established that *Giardia* sp., *Entamoeba* sp., *Blastocystis* sp., *Cryptosporidium* sp. and other parasitic infections can cause diarrhoea. Although they share similar clinical symptoms, it is important to note that the optimal approaches for their diagnosis, treatment and prevention can differ. Among them, *Blastocystis* sp. is one of the most common intestinal protozoans detected in humans (Kaewjai *et al*., 2023). Therefore, it is very important to establish a novel method for rapid and accurate diagnosis of *Blastocystis* sp.

At present, the main diagnostic methods for *Blastocystis* sp. are microscopic detection, immunological detection and molecular biological detection. The microscopic detection of *Blastocystis* sp. mainly includes fecal smear microscopy, staining microscopy and *in vitro* culture. However, the fecal smear method and staining microscopy are prone to false negatives, while *in vitro* culture is complicated and takes a long time to detect (Zamani *et al*., 2021). Immunofluorescence and enzyme-linked immunosorbent assay has low specificity and are currently limited to epidemiological and serological studies. Molecular biological diagnosis is one of the conventional diagnostic methods for *Blastocystis* sp. Conventional polymerase chain reaction (cPCR) has become increasingly common and popular in identifying species. This method has high sensitivity and specificity, especially in the differential diagnosis between species, and has also been used in the genotyping and differential diagnosis of *Blastocystis* sp. (Yoshikawa *et al*., 2004; Stensvold *et al*., 2006; Domínguez-Márquez *et al*., 2009; Khademvatan *et al*., 2018). However, cPCR requires particular equipment, including a thermal cycler, personnel with appropriate skills and training to conduct DNA-based diagnostic assays, and suitable cold storage to preserve the heat-sensitive reagents. This limitation hinders the widespread use and application in low-resource settings. Therefore, cPCR detection is usually only used in centralized clinical laboratories, highlighting the need for a faster, simpler and more accurate detection method that can be readily implemented in point of care settings.

Recombinase polymerase amplification (RPA), a isothermal amplification technique for nucleic acids, was developed in the UK (Piepenburg *et al*., 2006). It has been established that recombinases form nucleoprotein complexes with oligonucleotide primers and search for homologous sequences in the DNA in RPA reactions. Upon recognition of a specific homologous sequence, the RPA technique initiates the template's synthesis and then amplifies the reverse strand through isothermal chain replacement by polymerase, thus allowing amplification of double-stranded DNA without thermal or chemical denaturation. The RPA test can be done in an hour or less with a similar reagent cost to cPCR but with a lower labour cost and acceptable sensitivity and specificity levels (Mota *et al*., 2022). In recent years, this technique has been successfully applied to parasite diagnosis in many cases, including *Giardia duodenalis* (Crannell *et al*., 2015), *Entamoeba histolytica* (Nair *et al*., 2015) and *Leishmania Viannia spp.* (Saldarriaga *et al*., 2016). However, there is currently no RPA method available for the detection of *Blastocystis* sp.

This study aimed to develop an RPA detection method targeting the small subunit ribosomal RNA (*SSU rRNA*) gene of *Blastocystis* sp. and evaluate its sensitivity, specificity, stability and clinical efficacy.

## Materials and methods

### DNA sample used in this study

A total of 8 intestinal pathogens were used in this study, including *Blastocystis* sp., *Staphylococcus aureus*, *Escherichia coli*, *Dientamoeba fragilis*, *Trichuris trichiura*, *Ascaris lumbricoides*, *Giardia lamblia* and *Enterobius vermicularis.* Among them, the bacterial samples were from the strain bank of Pathogenic Biology, Xinxiang Medical University. *Blastocystis* sp., *D. fragilis*, *T. trichiura* and *G. lamblia* were isolated, cultured and enriched from feces. *Ascaris lumbricoides* and *E. vermicularis* were isolated directly from feces. The DNA of all pathogens was extracted by DNA extraction kit (Qiagen, Hilden, Germany) and identified by PCR amplification and sequencing. All identified DNA samples were stored at −20°C until used.

### Primer design

The RPA primers were designed according to the *SSU rRNA* gene sequence of *Blastocystis* sp. provided by the National Center for Biotechnology Information (NCBI), accession numbers: KR262943. The cPCR primers used in this study were obtained from a previous study by Scicluna *et al*. (2006). All primers were synthesized by Sangon Bioengineering Co., Ltd (Sangon, Shanghai, China). The sequence information of the primers is shown in Table 1.

**Table 1.** Primers used in this study

Assay	Primer name	Primer sequence (5’–3’)	Amplification size/bp
PCR	BhRDr	F: GAG CTT TTT AAC TGC AAC AAC G	600
	RD5	R: ATC TGG TTG ATC CTG CCA GT	
RPA	RPA-1-281-F	F: TCTTTATTAGTACCTTATGAGAAATCAAAGTCT	281
	RPA-1-281-R	R: GTTAAAGGCGGAGGTCTCGTTCGTTATCGG	
	RPA-2-175-F	F: TCCAGACATAGGAAGGATTGACAGATTG	175
	RPA-2-175-R	R: GTGTCCCTATAAGAAGCAACGCATATCA	
	RPA-3-232-F	F: TCCAGACATAGGAAGGATTGACAGATTG	232
	RPA-3-232-R	R: CATCTAAGGGCATCACAGACCTGTTATT	

F, forward; R, reverse.

### Primer screening for RPA assay

Three pairs of RPA primers designed in this study were selected for the RPA test, and the best pair of primers was screened according to the agarose gel electrophoresis results.

### Development of the RPA assay

The RPA assay was performed using the TwistAmp® Basic kit (TwistDx, Cambridge, UK) according to the manufacturer's instructions. Briefly, after the reaction mix (rehydration buffer, 29.5 *μ*L; forward primer, 2.4 *μ*L; reverse primer, 2.4 *μ*L; nuclease-free water, 8.2 *μ*L; 42.5 *μ*L) was prepared, it was added to a basic freeze-dried powder and thoroughly mixed. Subsequently, 5 *μ*L of DNA template and 2.5 *μ*L magnesium acetate solution were added to the suspension. The reaction tubes were incubated in a water bath at 37°C for 15 min. After the incubation, the amplified products were observed by agarose gel electrophoresis.

### Optimum reaction conditions for the RPA assay

The RPA assay was performed using the genomic DNA of *Blastocystis* sp. Seven reaction times (5, 10, 15, 20, 25, 30 and 35 min) and 7 temperatures (25, 30, 35, 37, 39, 41 and 45°C) were tested to determine the optimum reaction conditions for the RPA assay.

### The minimum detection limit of the RPA assay

The genomic DNA of *Blastocystis* sp. was subjected to a 10-fold gradient dilution to obtain concentrations of 100 ng *μ*L^−1^, 1 ng *μ*L^−1^, 10 pg *μ*L^−1^, 100 fg *μ*L^−1^, 1 fg *μ*L^−1^ and 10 ag *μ*L^−1^. Each diluted DNA sample (5 *μ*L) was used as a template and amplified using the RPA assay. In addition, cPCR was used to amplify the serially diluted DNA, and the minimum detection limit between RPA and cPCR was compared. For the cPCR amplification, a 25 *μ*L reaction system was prepared, containing 12.5 *μ*L of 2 × Taq Plus Master Mix, 8.5 *μ*L nuclease-free water, 1 *μ*L forward primer (10 *μ*M), 1 *μ*L reverse primer (10 *μ*M) and 2 *μ*L DNA. The thermal cycler (Thermo Fisher Scientific, Waltham, USA) settings were as follows: 94°C for 5 min (initial denaturation); 30 cycles at 94°C for 45 s (denaturation), 59°C for 1 min (annealing) and 72°C for 1 min (extension); 72°C for 10 min (final extension).

### Specificity of the RPA assay

For specificity analysis, the genomic DNA of *Blastocystis* sp. and 7 non-target pathogens (*S. aureus*, *E. coli*, *D. fragilis*, *T. trichiura*, *A. lumbricoides*, *G. lamblia* and *E. vermicularis*) were used as templates for the RPA and cPCR assays.

### Repeatability evaluation of the RPA assay

To confirm the repeatability of the developed RPA systems, genomic DNA at concentrations of 20, 4, 2 and 1 ng *μ*L^−1^ were used. Five independent RPA tests were performed on 5 days under the same conditions.

### Validation in clinical samples

The performance of the RPA assay to detect *Blastocystis* sp. was validated by 40 clinical fecal samples from the Third Affiliated Hospital of Xinxiang Medical University. The fecal samples were tested by the RPA assay and compared with the cPCR assay to ensure there were no instances of non-specific amplification.

## Results

### Primer screening for the RPA assay

**Figure 1.**
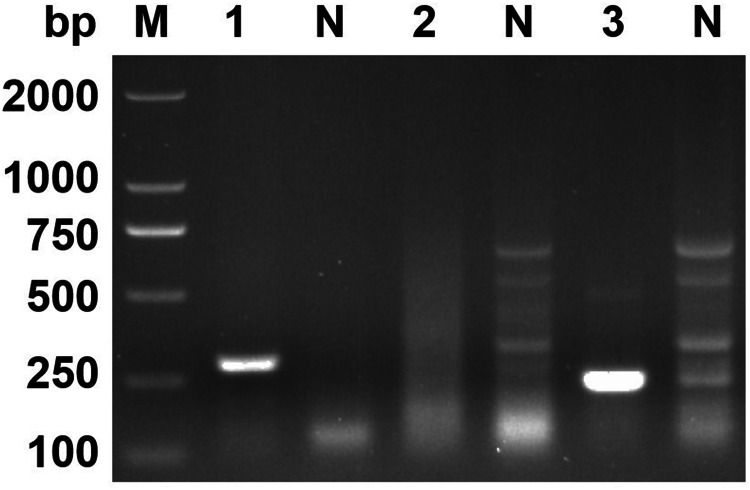
Primer screening. Three pairs of RPA primers were selected for the RPA test. Lane M: DL2000, lane 1: RPA-1-281-F/R, lane 2: RPA-2-175-F/R, lane 3: RPA-3-232-F/R, lane N: negative control.

The target genes amplified by 3 pairs of primers, RPA-1-281-F/R, RPA-2-175-F/R and RPA-3-232-F/R, were expected to have sizes of 281, 175 and 232 bp, respectively. The results were shown in Fig. 1. The target band was not amplified by RPA-2-175-F/R. Although RPA-3-232-F/R and RPA-1-281-F/R both obtained the correct target bands, only RPA-1-281-F/R was free of impurity bands. Therefore, RPA-1-281-F/R was chosen as the primer for RPA assay.

### Optimized RPA conditions

**Figure 2.**
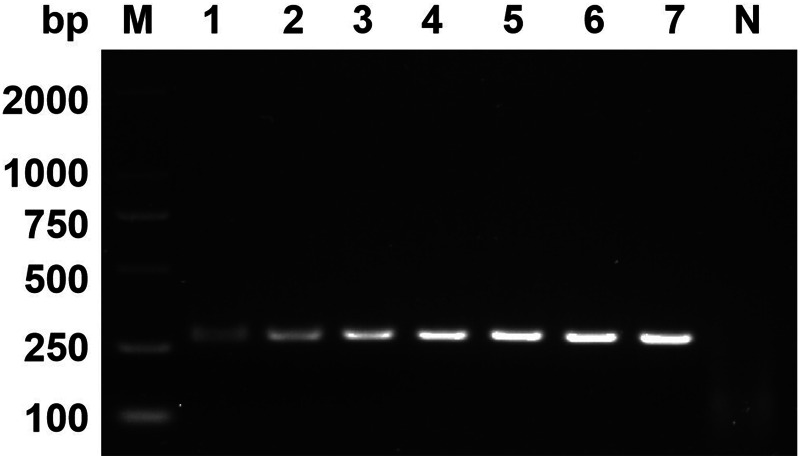
Optimum reaction time. Lane 1: 5 min, lane 2: 10 min, lane 3: 15 min, lane 4: 20 min, lane 5: 25 min, lane 6: 30 min, lane 7: 35 min, lane N: negative control.

**Figure 3.**
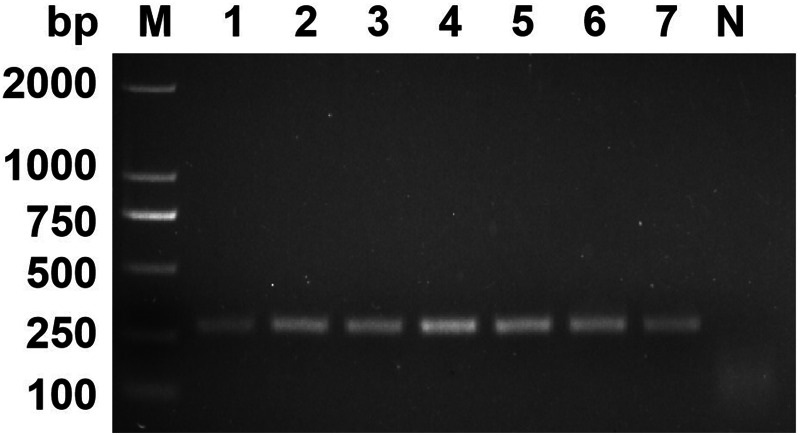
Optimum reaction temperature. Lane 1: 25°C, lane 2: 30°C, lane 3: 35°C, lane 4: 37°C, lane 5: 39°C, lane 6: 41°C, lane 7: 45°C, lane N: negative control.

The optimal RPA conditions were determined by the brightness of target bands on agarose gel electrophoresis. At an incubation temperature of 37°C, the target bands could be detected within 5 min. With increased incubation time, the brightness of the band increased. In this study, 30 min was selected as the final incubation time of the RPA assay (Fig. 2). When the incubation time was set as 30 min, the target bands were detected in the 25–45°C temperature range. Among them, 37°C had the highest amplification efficiency (Fig. 3).

### The minimum detection limit of the RPA assay

**Figure 4.**
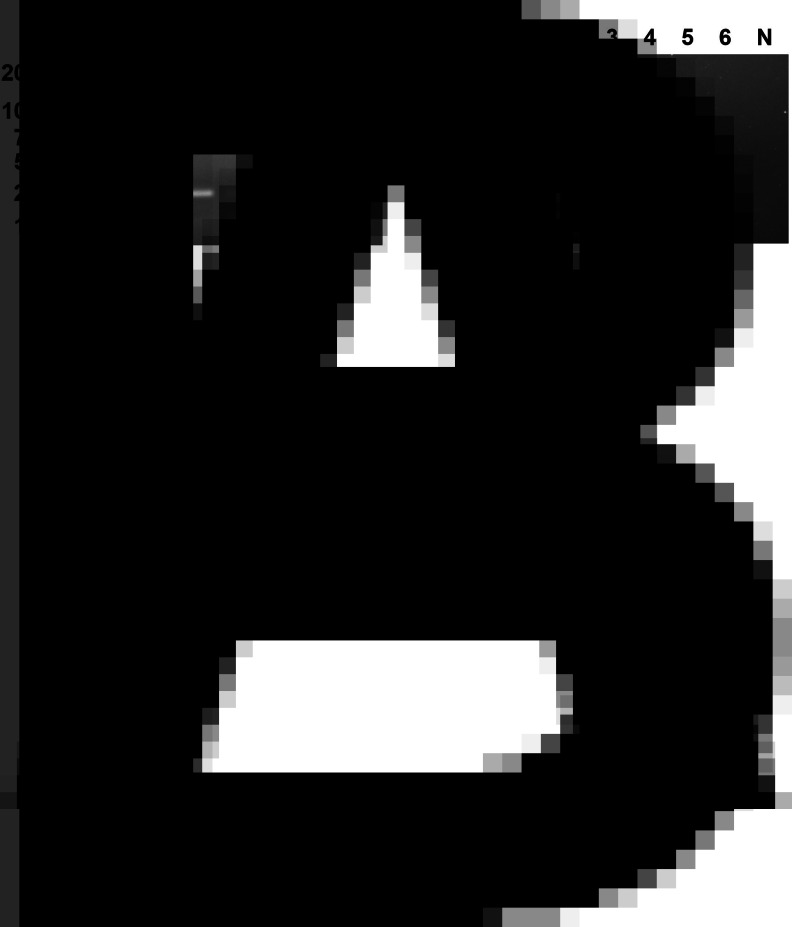
The minimum detection limit of RPA assay. Different concentrations of DNA were used for the RPA assay and cPCR assay. (A) The minimum detection limit of RPA assay. (B) The minimum detection limit of cPCR assay. Lane 1: 100 ng *μ*L^−1^, lane 2: 1 ng *μ*L^−1^, lane 3: 10 pg *μ*L^−1^, lane 4: 100 fg *μ*L^−1^, lane 5: 1 fg *μ*L^−1^, lane 6: 10 ag *μ*L^−1^, lane N: negative control.

Six genomic DNA dilutions were detected by the RPA and cPCR assays, respectively, and the results are shown in Fig. 4. The minimum detection limit of RPA and cPCR assays was 100 fg *μ*L^−1^, indicating that the detection method established in this study had high sensitivity.

### Specificity of the RPA assay

**Figure 5.**
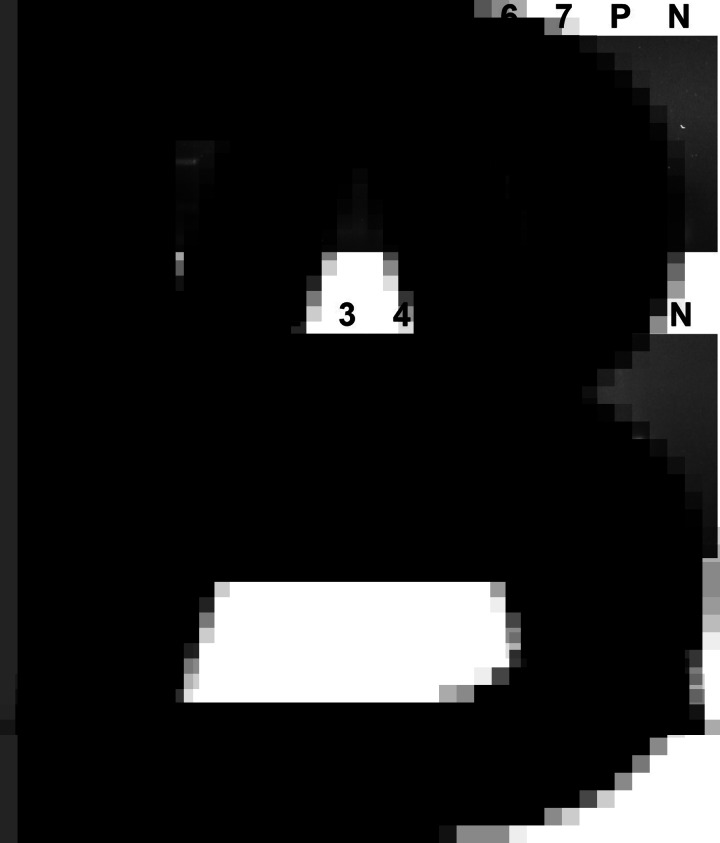
Specificity analysis. The genomic DNA of *Blastocystis* sp. and 7 non-target pathogens were used as templates for RPA assay and cPCR assay. (A) Specificity analysis of RPA assay. (B) Specificity analysis of cPCR assay. Lane 1: *Enterobius vermicularis*, lane 2: *Ascaris lumbricoides*, lane 3: *Dientamoeba fragilis*, lane 4: *Trichuris trichiura*, lane 5: *Giardia lamblia*, lane 6: *Escherichia coli*, lane 7: *Staphylococcus aureus*, lane P: positive control, lane N: negative control.

As shown in Fig. 5, *Blastocystis* sp. and 7 other non-target pathogens could be accurately distinguished by the RPA assay. The specificity of the RPA assay was consistent with the cPCR assay, indicating that the RPA assay has high specificity.

### Repeatability evaluation of the RPA assay

As shown in Table 2, the 5 test results were completely consistent. Besides, there was no false-negative or false-positive result, indicating the good repeatability of the RPA assay.

**Table 2.** Repeatability evaluation of RPA assay

Results	The genomic DNA of *Blastocystis* sp.				ddH_2_O
	10-fold dilution	50-fold dilution	100-fold dilution	200-fold dilution	
Number of positives	5	5	5	5	0
Number of negatives	0	0	0	0	5

### Performance validation with clinical samples

**Figure 6.**
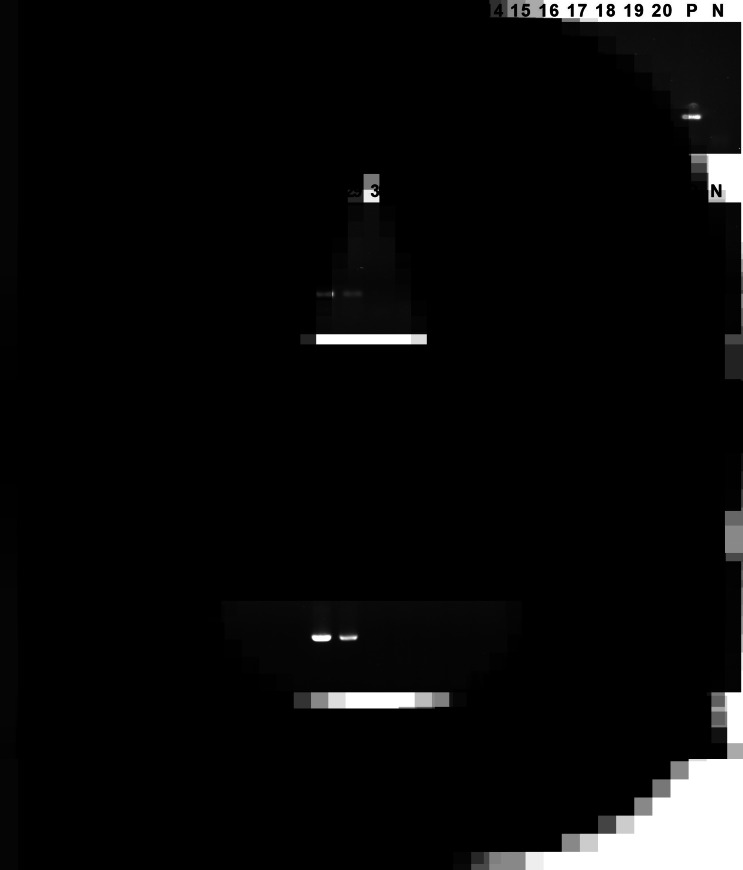
Performance validation with clinical samples. The 40 clinical fecal samples were tested by RPA assay and cPCR assay to check for any non-specific amplification. (A) and (B) were RPA assays. (C) and (D) were cPCR assays. Lane 1–40: clinical fecal samples, lane P: positive control, lane N: negative control.

Forty clinical samples were tested by the RPA and cPCR assays, respectively (Fig. 6). The RPA test identified that 9 samples were positive for *Blastocystis* sp., and 31 were negative. These results were consistent with cPCR results, indicating high sensitivity and specificity of RPA assay. Therefore, the RPA assay proved to be a highly effective method for detecting *Blastocystis* sp.

## Discussion

It has been established that *Blastocystis* sp. exhibits significant genetic diversity in the *SSU rRNA* gene, which allows all the subtypes to be identified and can be used for large-scale epidemiological investigations (Scicluna *et al*., 2006). Therefore, the *SSU rRNA* gene of *Blastocystis* sp. was selected as the target sequence in this study. The results showed that the RPA method could detect *Blastocystis* sp., and there was no cross-reaction with 7 non-target pathogens in the gut, indicating that the method had good specificity. The minimum detection limit test results showed that the minimum detection limit of *Blastocystis* sp. genomic DNA was 100 fg *μ*L^−1^, consistent with the minimum detection limit of the cPCR assay. The RPA assay could be carried out at 25–45°C, indicating its lower temperature requirements and wider detection range, conducive to the detection of *Blastocystis* sp. in low-resource settings (Abd *et al*., 2015). In addition, the target bands appeared after a reaction time of 5 min. When the reaction time was 30 min, the target bands became clearer, suggesting the optimal reaction time was 30 min. Compared with traditional detection methods or general molecular detection techniques (such as cPCR or loop-mediated isothermal amplification), the RPA assay significantly reduces detection time, leading to improved timeliness of results (Fang *et al*., 2011; Herrero *et al*., 2011).

Unlike PCR, which includes a high-temperature denaturation step to prevent non-specific amplification, the isothermal nature of RPA reactions makes them more susceptible to the formation of primer dimers. Primer dimers are unintended products formed when primers anneal to each other instead of the target sequence, leading to reduced amplification efficiency and may affect the test results in case of low or absent template concentration (Özay and McCalla, 2021). Therefore, 3 RPA primers were designed with the *SSU rRNA* gene as the target sequence in this study. RPA-1-281-F/R was selected by agarose gel electrophoresis as the best primer pair for detecting *Blastocystis* sp.

Higher sensitivity increases the likelihood of false-positive results during molecular biological detection. Therefore, 5 independent repeatable experiments were performed in this study, and the results showed that the RPA method established in this study ensured sensitivity while preventing false positives by optimizing the design of primers, reaction conditions and the entire reaction system. Meanwhile, the results of 40 clinical samples were consistent with the results of cPCR, indicating that the results of this method are highly credible and have good application prospects.

In summary, we constructed an isothermal amplification method for *Blastocystis* sp. nucleic acid using RPA technology. This method offers advantages, such as faster detection, simplicity in execution and high sensitivity and specificity rates, making it highly applicable for rapid *Blastocystis* sp. detection.

## Data Availability

The datasets supporting the findings of this article are included within the article and its additional files.
